# Insects as an emerging and alternative ingredient of feed for domestic animals

**DOI:** 10.5713/ab.2022.0001ED

**Published:** 2022-02-22

**Authors:** Cheol-Heui Yun

**Affiliations:** 1 Department of Agricultural Biotechnology and Research Institute of Agriculture and Life Sciences, Seoul National University, Seoul 08826, Korea; 2 Co Editor-in-Chief, Animal Bioscience, Seoul 08776, Korea

Hunger, poverty and disease, called triple burden of malnutrition, are the enemy of mankind and often cause of losing the faith in humanity. On the other hand, the global population is estimated to continuously grow until 2050, reaching over 9 billion people. The search for new food solutions with a good nutritional value for direct and indirect human consumption is of fundamental importance. Needless to say, we need 70% more food in which the livestock production continues to be indispensable as a protein source to make sure that per capita intake is being properly maintained. A real question is whether we are able to provide enough animal feed to meet the global capacity and the quest for novel feed resources is a must.

The replacement and/or alternatives of cost-effective and eco-friendly resources in food and feed production is imperative. Insect rearing could be one of the ways to solve the problem, and at the same time to enhance food and feed security. The use of insects for feeding farmed animals might be a promising alternative for not only their nutritional values but also possible environmental benefits, given the circular economy and sustainability. They grow and reproduce relatively easily, have high feed conversion efficiency and can be reared on bio-waste streams. Indeed, they can be reared sustainably and mass-produced on food production residues, and contribute in a positive circular economy that dramatically diminishes or even eliminates food- and feed-waste through bioconversion. Therefore, insects have been investigated and used as human food and as feed for pigs and birds, aquatic animals, and ruminant. Recently, InnovaFeed, a French biotech insect producer for farmed animal nutrition, announced an agreement to supply insect protein for pet food at annual production capacity of 60,000 tons derived from black soldier flies when production is scaled up by 2024 (https://www.world-grain.com/articles/16438-innovafeed-to-supply-insect-protein-to-adm).

With such atmosphere and swift changes on global market to meet the requirement in the field of farmed animals, Animal Bioscience (AB) hosted Animal Bioscience Forum 2021, sponsored by Pathway Intermediates, Korea. The Forum has taken place through a virtual conference (Webinar), because of pandemic situation, on September 28–29, 2021. The presentation in a video format and review papers are freely available at the Home page (https://www.animbiosci.org/) where one can find a valuable information about potential use of variety of insects for farmed animals including pigs, chickens, and ruminant.

Shah et al [1] reviewed nutritional composition and value of insects including black soldier flies, grasshoppers, mealworms, housefly larvae, and crickets, in order to examine their potential use as an alternative protein source for feed ingredient in domestic animals and fishes. The authors discussed the fact from the related articles that various insects and their by-products could promote and support sustainable animal production since they are composed of valuable nutrients not only protein but also lauric acid, essential fatty acids, and bioactive compounds, such as chitin.

Elahi et al [2] introduced a wide range of insects including black soldier fly, mealworm, housefly, cricket/grasshopper/locust (Orthoptera), silkworm, and earthworm for potential use as feed ingredients in poultry because of rich essential amino acids and antimicrobial peptides when compared with conventional feedstuffs. The expectation is to improve the growth performance, nutrient digestibility, intestinal health, and immune function in poultry. Authors addressed benefits of insect meal in poultry diet accordance with the main challenge for its proper utilization and emphasized the need for their mode of action studies to reveal their functional aspect.

Hong and Kim [3] suggested black soldier fly (*Hermetia illucens*), yellow mealworm (*Tenebrio molitor*), and common housefly (*Musca domestica*) as feed ingredients and an alternative protein source for pigs. They have pointed out that each ingredient may have different effects for growth performance, nutrient digestibility and importantly pork quality dependent on the phase feeding plan for pigs. Thus, the authors expected further studies on the optimal inclusion level of insect products in whole range of animals from weaned piglets to sow.

Astuti and Wiryawan [4] reviewed unique, yet necessary, studies focusing black soldier fly (BSF) and its by-product as a feed ingredient for ruminants. Intriguingly, a number of studies suggested potential use of BSF larvae and BSF frass as not only milk replacer, creep feed and substitution for soybean meal but also prebiotics for lactic acid bacteria.

Collectively, while the investigation aims further to elucidate the exact mode of action of the insects and their byproducts with proper concentration as a source of ingredient for animal feed, there are several issues yet to be solved as following; i) differences among different animal species including pets and aquatic fishes, ii) their absorption and digestibility in relation to growth promotion, iii) interaction with microbiota, iv) effects on microbiota and metabolites that influence growth of the animals, v) waste and risk assessment, vi) large scale production, vii) break-even point for commercial sector in relation with cost-effectiveness and a profit, viii) environmental impact, ix) regulatory issues for not only process of the ingredients, quality of end product but also national and international trade.
